# Supplementary material for: Splice-Junction-Based Mapping of Alternative Isoforms in the Human Proteome
Source: Cell Rep. Author manuscript; Available in PMC 2020 Jan 15. (PMC6961840; doi:10.1016/j.celrep.2019.11.026)
Supplement: 2 [file NIHMS1546469-supplement-2.zip › DF1/PXD010154/Lung/Lung_25_PDCD6_YIPGAGPGPAAGAALPDQSFLWNVFQR.pdf]

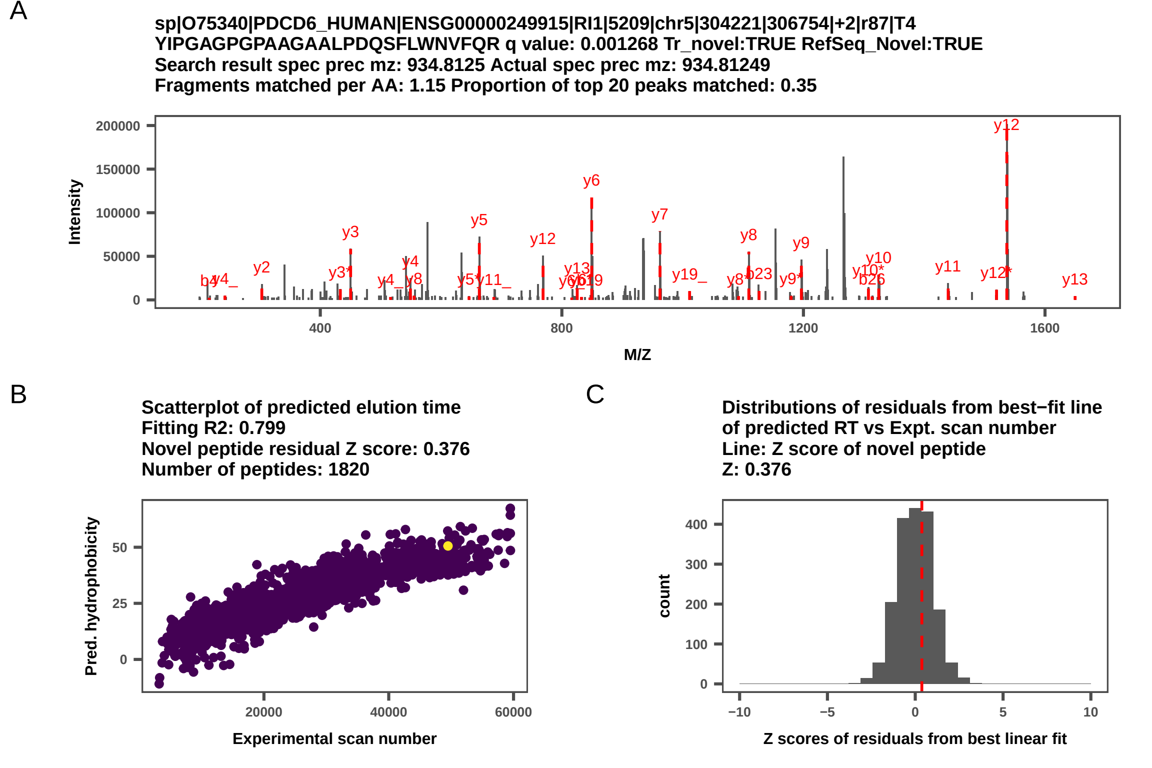
A
sp|O75340|PDCD6_HUMAN|ENSG00000249915|RI1|5209|chr5|304221|306754|+2|r87|T4
YIPGAGPGPAAGAALPDQSFLWNVFQR q value: 0.001268 Tr_novel:TRUE RefSeq_Novel:TRUE
Search result spec prec mz: 934.8125 Actual spec prec mz: 934.81249
Fragments matched per AA: 1.15 Proportion of top 20 peaks matched: 0.35
Intensity
200000
150000
100000
50000
0
400
800
1200
1600
M/Z
y12
y6
y7
y5
y3
y12
y8
y9
y10
y11
y13
y2
B
Scatterplot of predicted elution time
Fitting R2: 0.799
Novel peptide residual Z score: 0.376
Number of peptides: 1820
Pred. hydrophobicity
50
25
0
20000
40000
60000
Experimental scan number
C
Distributions of residuals from best−fit line
of predicted RT vs Expt. scan number
Line: Z score of novel peptide
Z: 0.376
count
400
300
200
100
0
−10
−5
0
5
10
Z scores of residuals from best linear fit
